# A pilot randomized aerobic exercise trial in older HIV-infected men: Insights into strategies for successful aging with HIV

**DOI:** 10.1371/journal.pone.0198855

**Published:** 2018-06-12

**Authors:** Krisann K. Oursler, John D. Sorkin, Alice S. Ryan, Leslie I. Katzel

**Affiliations:** 1 Geriatric Research, Education, and Clinical Center, Baltimore Veterans Affairs Medical Center, Veterans Affairs Maryland Health Care System, Baltimore, MD, United States of America; 2 Department of Medicine, Division Gerontology and Geriatric Medicine, The University of Maryland School of Medicine, Baltimore, MD, United States of America; Rush University, UNITED STATES

## Abstract

**Background:**

HIV-infected adults have increased risk for age-related diseases and low cardiorespiratory fitness that can be prevented and improved with exercise. Yet, exercise strategies have not been well studied in older adults with HIV and may require substantial adaptation to this special population.

**Objective:**

To determine the safety and efficacy of aerobic exercise in older HIV-infected men in a randomized trial comparing different levels of exercise intensity.

**Methods:**

We conducted a pilot exercise trial in 22 HIV-infected men ≥50 years of age receiving antiretroviral therapy who were randomized 1:1 to moderate-intensity aerobic exercise (Mod-AEX) or high-intensity aerobic exercise (High-AEX) that was performed three times weekly for 16 weeks in a supervised setting. Primary outcome was cardiorespiratory fitness (VO_2_peak) measured by treadmill testing. Secondary outcomes were exercise endurance, six-minute walk distance (6-MWD), body composition measured by Dual-energy X-ray absorptiometry (DXA), and fasting plasma levels of lipids and glucose.

**Results:**

VO_2_peak increased in the High-AEX group (3.6 ±1.2 mL/kg/min, p = 0.02) but not in the Mod-AEX group (0.4 ±1.4 mL/kg/min, p = 0.7) with a significant between group difference (p<0.01). Exercise endurance increased in both the High-AEX group (27 ±11%, p = 0.02) and the Mod-AEX group (11 ±4%, p = 0.04). The 6-MWD increased in both the High-AEX (62 ±18m, p = 0.01) and the Mod-AEX group (54 ±14m, p = 0.01). Changes in VO_2_peak and 6-MWD were clinically relevant. There were no serious exercise-related adverse events. Dropouts were similar between group (27% overall) and were related to joint pain.

**Conclusions:**

This pilot exercise trial demonstrates that moderate to high-intensity aerobic exercise in older HIV-infected men increases endurance and ambulatory function. However, increased cardiorespiratory fitness was observed only with high-intensity aerobic exercise despite substantial baseline impairment. Future research is needed to determine exercise strategies in older HIV-infected adults that address advanced aging and comorbidity yet are durable and feasible.

## Introduction

While the median survival time for HIV-infected adults 50 years of age and older has doubled, it remains lower than the general population.[1] Despite advances in antiretroviral therapy, HIV-infected adults have an increased risk for age-related diseases and syndromes (e.g., frailty and falls) that is independent of traditional risk factors.[2] This advanced aging phenotype has led to new clinical care challenges for older HIV-infected adults [3,4] who represent more than half of HIV-infected adults in the United States.[5]

In older adults without HIV the positive impact of exercise on cardiovascular disease,[6] frailty, and falls [7] is well established. However, exercise strategies may require substantial adaptation to older HIV-infected adults to counteract the advanced primary aging process, e.g., inflammation and mitochondrial dysfunction. VO_2_peak, oxygen utilization at peak exercise, is an established measure of cardiorespiratory fitness and a physiologic biomarker of aging that declines in sedentary adults 10% per decade [8] and independently predicts cardiovascular and all-cause mortality.[9,10] Cardiorespiratory fitness is substantially reduced in HIV-infected adults [11,12] and correlates with biomarkers of inflammation[13] and skeletal muscle mitochondrial function.[14] Results from exercise interventions in older adults with multimorbidity, but not HIV infection, shows that high-intensity aerobic exercise is necessary to decrease plasma levels of inflammation.[15]

Although moderate to high-intensity aerobic exercise increases VO_2_peak in younger HIV-infected adults, the effect and safety, in older HIV-infected adults is understudied.[16,17] To address this gap, we conducted a pilot randomized aerobic exercise trial that compared the effect of moderate-intensity versus high-intensity aerobic exercise on VO_2_peak in older HIV-infected men. We hypothesized that aerobic exercise would increase VO_2_peak in both groups but high-intensity exercise would be poorly tolerated. The aim of the trial was to collect preliminary efficacy and safety data toward the goal of determining the ideal exercise program in HIV-infected adults that targets mechanisms of advanced aging.

## Methods

### Study design

Sedentary HIV-infected adults ≥50 years of age were recruited from the Baltimore VA Medical Center (VAMC) HIV Clinic between 2008 and 2011. After completion of screening and baseline testing participants were randomized 1:1 to 16 weeks of moderate-intensity aerobic exercise training (Mod-AEX) or high-intensity aerobic exercise training (High-AEX). The randomization sequence was generated using a computer program by an investigator without participant contact (JS) and distributed using the closed envelope method to the research coordinator. The study was approved by the University of Maryland School of Medicine Institutional Review Board and the Baltimore VAMC Research and Development Committee. Sample size target was based on a randomized trial in 21 younger HIV-infected men that compared the effect of moderate-intensity and high-intensity aerobic training on endurance (time on treadmill)[18] since no comparably designed trial in the HIV literature measured VO_2_peak.

### Participants

Eligibility criteria included a stable combination antiretroviral therapy (cART) and no AIDS defining conditions in the prior six months. Participants were excluded for conditions that increase the risk of exercise training per the American College of Sports Medicine (ACSM), which included, but was not limited to, poorly controlled blood pressure, symptoms of angina and claudication, and ambulation with an assistive device.[19] Participants that had evidence of ischemic heart disease on baseline treadmill testing were excluded. Participants with moderate to severe anemia (HGB <10) were excluded as an additional safety precaution and potential source of confounding based on our prior work in this patient population.[11] Use of a beta-blocker or non-dihydropyridine calcium channel blocker was also an exclusion criterion because it affects heart rate which was monitored for exercise training fidelity. All participants provided written informed consent. The study was registered retroactively at ClinicalTrials.gov (NCT03399136) after completion in 2012 since it preceded FDAAA 801 requirements. The authors confirm that all ongoing and related trials of exercise training in HIV-infected adults are registered (NCT02101060).

### Exercise intervention

Participants attended center-based exercise sessions three times per week at the research exercise training facility located at the Baltimore VA Medical Center. All exercise training was performed under the supervision of research exercise physiologists. Heart rate (HR) was measured continuously during each session using a Polar HR watch and chest strap allowing for assessment and maintenance of treatment fidelity. Exercise logs included details on exercise type, duration, time in target heart rate zone, blood pressure and perceived intensity during each training session. In the High-AEX group, exercise training was performed on a motorized treadmill with occasional substitution with the elliptical machine as needed for joint pain. Target heart rate was based on the baseline treadmill test and was calculated as percentage of the heart rate reserve (HRR = maximal HR-resting HR). Initially, participants trained for 20–30 minutes at 50–60% of HRR. Duration and intensity was increased by 10% weekly so that within 5–7 weeks the aerobic exercise sessions lasted 30–45 minutes at 70–85% of HRR and at the end of the 16 weeks lasted 40–45 minutes at 75–90% of HRR. In the Mod-AEX group, participants performed a self-paced 1-mile walk (3–5 METs) on an indoor track in the same exercise center as the high-AEX group. Initial sessions lasted 20–30 minutes and were increased weekly to 45 minutes in parallel to the duration of the high-AEX group. Participants were encouraged to stay weight neutral and had their weight and diet routinely assessed to meet that goal. Adverse events were evaluated by an investigator blinded to group allocation.

### Outcomes

#### Cardiorespiratory fitness

The primary outcome measure of the study was cardiorespiratory fitness (oxygen utilization at peak exercise, VO_2_peak) that was measured during a graded exercise test on a treadmill using the modified Bruce protocol as defined by the ACSM.[19] A cardiologist blinded to participant information reviewed the continuous ECG tracing for evidence of ischemia per Minnesota Criteria. Breath-by-breath oxygen utilization, carbon dioxide production, and minute ventilation values were averaged at rolling 10-second intervals using a Sensor Medics Vmax 29C series metabolic cart. VO_2_peak was calculated as the average of the highest two oxygen utilization values at the end of exercise. Exercise duration was measured as total exercise time on the treadmill.

#### Six-minute walk distance

The distance covered during a six minute walk test, a commonly used measure of functional capacity, was conducted according to the guidelines of the American Thoracic Society [20] at least 48 hours from the treadmill test.

#### Body composition

Dual-energy X-ray absorptiometry (DXA) (Model GE iDXA Scan) was used to measure total and regional lean tissue and fat mass. Computed tomography (CT) scans (PQ 6000 Scanner, GE Medical Systems) at the level of L4-L5 were performed to determine the amount of visceral and subcutaneous fat area of the abdomen. Anthropometric measures included waist and hip circumference. Body mass index (BMI) was calculated as weight/(height)^2^. Participants were classified with lipodystrophy based on a fat mass ratio ≥1.26 (% trunk fat: % leg fat).[21]

#### Metabolic disease

Participants had blood drawn after an overnight fast. Plasma glucose was measured by glucose oxidase method (YSI, Yellow Springs, OH) and insulin by double antibody radioimmunoassay (Millipore, St. Charles, MO). Plasma triglycerides and cholesterol were analyzed by enzymatic methods (Unicel DxC880i; Beckman Coulter, Inc., Brea, CA). HDL-C concentration was measured in the supernatant after precipitation with dextran sulfate. LDL-C concentration was computed using the Friedewald equation.[22]

#### Clinical measures

Additional data on clinical characteristics were obtained from the VA electronic medical record (EMR) including medical history, laboratory and pharmacy data, which were confirmed by participant self-report at enrollment. Additional blood testing included CD4 cell count and HIV-1 RNA plasma level (Abbott Real Time HIV-1, Abbott Molecular Inc., Des Plaines, IL). Smoking and injection drug use were assessed by self-report. Diagnosis of hypertension and diabetes was based on clinical history.

#### Statistical analysis

Descriptive statistics were used to examine data for distribution. Between group differences in characteristics at baseline were evaluated with Student’s t-test, Wilcoxon rank-sum test, and Pearson’s Chi squared test. Within group changes (post-pre intervention) were examined using a paired Student’s t-test or Wilcoxon signed rank test. Between group differences in outcome change were examined using ANCOVA in which the outcome change was the dependent variable in a model adjusted for the baseline value of the outcome variable and group (Mod-AEX vs. High-AEX). Analyses were performed using STATA software (v11, StataCorp, College Station, TX).

## Results

**Fig 1:**
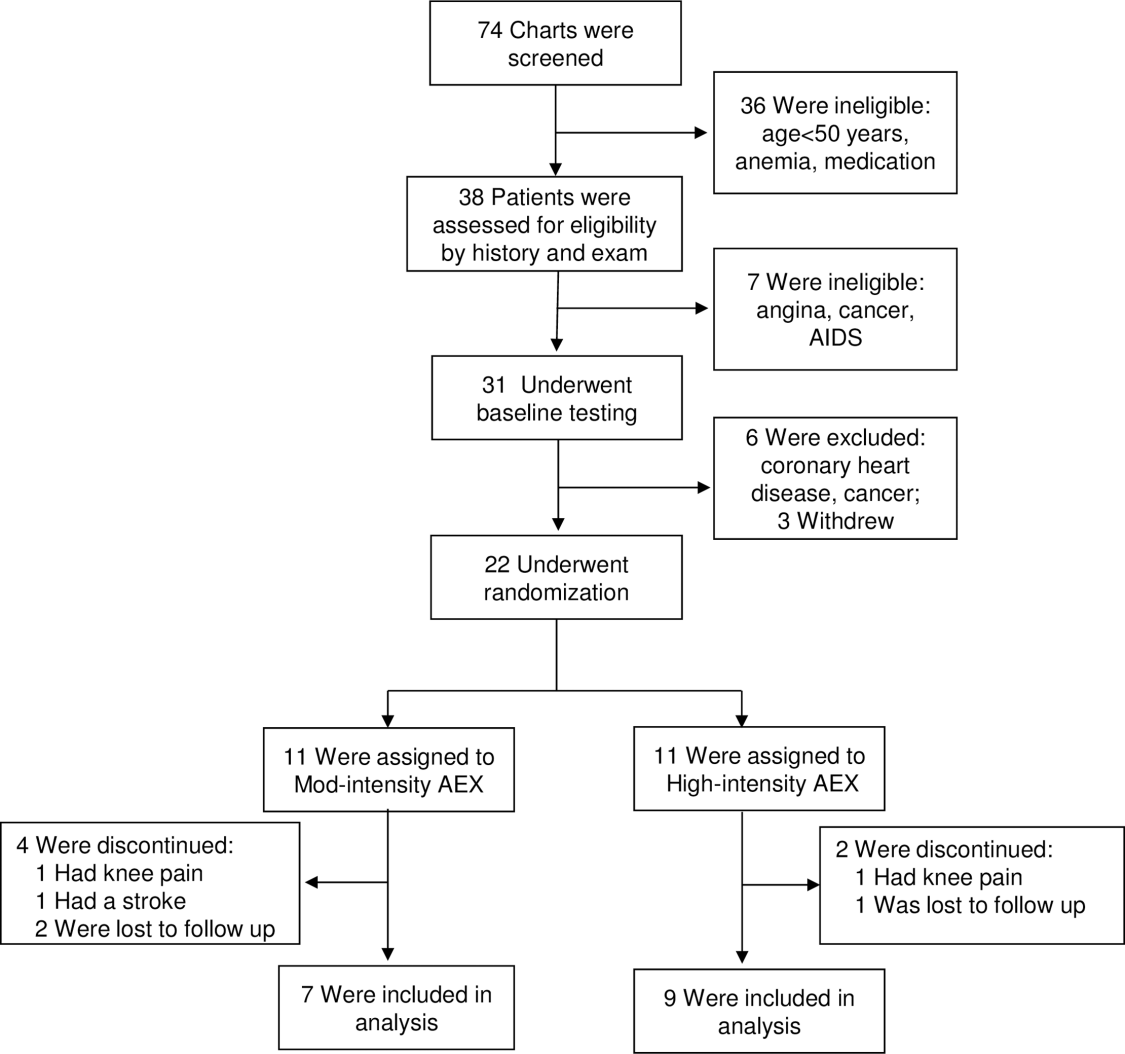
Trial profile.

The trial overview is provided in Fig 1. Chart review of the VA electronic medical record was performed in 74 HIV-infected patients and screened out individuals based on criteria for age <50 years or medication (cART and cardiac drugs). All individuals were male, consistent with the target patient population of older Veterans. Thirty-eight interested participants provided written informed consent and underwent a history and physical exam. Seven participants were excluded after the eligibility history and physical exam based on existing conditions. During baseline testing, 6 participants were excluded and 3 participants dropped out. Twenty-two participants were randomized and represent the analytical set. Baseline characteristics for participants by group are summarized in Table 1. Despite randomization, the Mod-AEX group had a higher body weight and greater adiposity. During the 16-week intervention, 4/11 participants in the Mod-AEX group and 2/11 in the High-AEX group dropped out due to osteoarthritis (n = 2) or stroke at home (n = 1), or were lost to follow-up (n = 3) for an attrition rate of 27%. The primary adverse event was joint pain which occurred in approximately a quarter of participants in both groups but limited exercise in only 2 individuals (Fig 1). Exercise training was otherwise well-tolerated. There were no serious exercise-related adverse events in either exercise training groups. Compliance to training visits was excellent among those who completed the intervention; the mean attendance rate was 89%, which did not vary by group.

**Table 1:** Characteristics of the study population randomized to high-intensity or moderate-intensity exercise group.

Characteristic		High Intensity n = 9	Moderate Intensity n = 7
**Age, years, mean (SD)**		57.4 (5.6)	57.4 (3.9)
**Race, n (%)**			
White		2 (22%)	0 (0%)
Black		7 (78%)	6 (86%)
Hispanic		0 (0%)	1 (14%)
**HIV characteristics and antiretroviral therapy**			
CD4, cells/ml, mean (SD)		481.2 (196.8)	469.3 (251.2)
<75 copies HIV-1 RNA, n (%)		8 (89%)	7 (100%)
History of AIDS defining illness, n (%)		2 (22%)	5 (71%)
Nucleoside Reverse Transcriptase Inhibitor(s), n (%)		9 (100%)	9 (100%)
Boosted Protease Inhibitor, n (%)	6 (67%)		7 (100%)
Efavirenz, n (%)	3 (33%)		0 (0%)
**Weight and body composition, mean (SD)**			
Weight, kg	77.2 (16.2)		97.6 (8.8)*
Body Mass Index, kg/m^2^	25.6 (5.3)		32.6 (3.0)*
Whole Body DXA:			
Lean mass, kg	58.6 (11.4)		59.0 (4.5)
Fat mass, kg	17.1 (7.8)		31.3 (4.6)*
Body fat, percent	21.3 (6.7)		33.9 (4)*
Abdominal CT Scan:			
Visceral fat area, cm^2^	83.6 33.4)		180.5 83.1)*
Subcutaneous fat area, cm^2^	174.4 (71.0)		394.6 (68.6)*
Lipodystrophy, n (%)	5 (56%)		5 (71%)
**Metabolic parameters, mean (SD)**			
Total Cholesterol, mg/dl	157 (49.9)		157 (34.4)
HDL-C, mg/dl	47 (15.8)		40 (3.78)
LDL-C, mg/dl	87 (38.4)		80 (28.9)
Triglycerides, mg/dl, median (IQR)	105 (85–152)		145 (100–185)
Fasting glucose, mmol/l, median (IQR)	103 (90–134)		102 (93–169)
Fasting insulin, pmol/l	67(36.0)		154 (45.5)*
**Comorbidities**			
Hypertension, n (%)	4 (44%)		4 (57%)
Diabetes, n (%)	2 (22%)		3 (43%)
Hepatitis C, n (%)	3 (33%)		3 (43%)
**Health Behaviors**			
Smoker, current, n (%)	8 (89%)		4 (57%)
Substance Abuse			
History of alcohol disorders, n (%)	2 (22%)		3 (43%)
History of injection drug use, n (%)	4 (44%)		1 (14%)

Lipodystrophy, fat mass ratio ≥1.26 (% trunk fat/% leg fat) [21].

Compared by chi-squared test; Student’s t-test or Wilcoxon rank-sum test (triglycerides, glucose).

* p <0.05.

Training effects are summarized in Table 2. VO_2_peak increased in the High-AEX group (3.6 ±1.2 mL/kg/min, p = 0.02) but not in the Mod-AEX group (0.4 ±1.4 mL/kg/min, p = 0.7) with a significant between group difference. Time on treadmill significantly increased in both the Mod-AEX (11%) and High-AEX (27%) groups. The six-minute walk distance significantly increased 11% in both the Mod-AEX and High-AEX groups. Weight remained stable (<0.5 kg change) in both groups. Body composition and metabolic parameters did not significantly change in either group. However, the between group difference in change for HDL-C was statistically significant with a clinically relevant increase in the High-AEX group and minor decrease in the Mod-AEX group.

**Table 2:** Effect of aerobic exercise training by intensity on exercise capacity, body composition, and metabolic parameters.

	High Intensity (n = 9)				Moderate Intensity (n = 7)				
Outcome	Baseline	16-week	Change	p-value within group*	Baseline	16-week	Change	p-value within group*	Adjusted mean (95% CI) of change between groups**
**Exercise capacity**									
VO_2_peak, ml/kg/min	23.8 ±1.2	27.4 ±1.2	3.6 ±1.2	0.02	17.8 ±1.8	18.2 ±1.0	0.4 ±1.4	0.78	6.6 (3.1,10.2)
VO_2_peak, L/min	1.89 ±0.19	2.12 ±0.15	0.23 ±0.08	0.03	1.73 ±0.17	1.75 ±0.12	0.02 ±0.16	0.92	0.3 (0.0, 0.5)
Time on treadmill, mins	11.7 ±0.8	14.3 ±0.6	2.6 ±0.8	0.02	11.0 ±0.5	12.1 ±0.5	1.1 ±0.4	0.04	1.9 (0.5, 3.4)
6-MWD, meter	551 ±33	613 ±36	62 ±18	0.01	486 ±19	540 ±19	54 ±14	0.01	14.9 (-36.6, 66.3)
**Weight and body composition**									
Weight, kg	77.2 ±5.4	77.9 ±5.1	0.7 ±0.9	0.48	97.6 ±3.3	95.8 ±3.9	-1.8 ±2.0	0.40	1.6 (-3.7, 6.9)
Whole Body DXA									
Lean mass, kg	58.6 ±3.8	58.4 ±3.7	-0.2 ±0.6	0.80	59.0 ±2.0	58.6 ±1.9	-0.4 ±1.3	0.76	0.3 (-2.3, 2.8)
Fat mass, kg	17.2 ±2.6	17.2 ±2.4	0.05 ±0.4	0.92	31.3 ±2.1	31.1 ±2.2	-0.2 ±1.9	0.91	-1.4 (-5.8, 3.0)
Body fat, percent	21.3 ±2.4	21.6 ±2.1	0.3 ±0.6	0.67	33.9 ±2.0	33.6 ±1.4	-0.3 ±1.4	0.85	-2.1 (-5.5, 1.4)
Abdominal CT Scan									
Visceral fat area, cm^2^	84 ±13	92 ±16	8 ±7	0.25	181 ±34	187 ±30	6 ±18	0.75	-16 (-62, 30)
Subcutaneous fat area, cm^2^	174 ±27	170 ±26	-5 ±8	0.57	395 ±28	381 ±35	-14 ±16	0.44	10.3 (-59.2, 79.8)
**Metabolic parameters**									
Total Cholesterol, mg/dl	157	174	16	0.39	157	163	5	0.15	11.1 (-19.7, 41.9)
HDL-C, mg/dl	47	56	9	0.25	40	39	-1	0.63	16.0 (2.4, 29.7)
LDL-C, mg/dl	87	96	9	0.42	80	82	2	0.56	9.4 (-15.0, 33.9)
Triglycerides, mg/dl	117	109	-8	0.57	221	227	5	0.86	-36.9 (-77.3, 3.5)
Fasting glucose, mmol/l	107	110	3	0.60	133	104	-29	0.28	19.9 (-22.6, 62.3)

Values are mean ± SE. CT scan: N = 13 (7 high-AEX, 6 mod-AEX)

*paired t-test or Wilcoxon signed rank sum (triglycerides).

** ANCOVA includes adjustment for baseline values.

## Discussion

This pilot randomized exercise trial supports the safety and efficacy of aerobic exercise training in older HIV-infected men. In both the moderate-intensity and high-intensity groups we found clinically significant increases in exercise endurance and six-minute walk distance. Converse to our hypothesis, high-intensity aerobic exercise was well-tolerated and resulted in a clinically and statistically significant increase in cardiorespiratory fitness (VO_2_peak) that was not observed in the moderate intensity group.

Several reviews and meta-analyses of randomized exercise trials in younger HIV-infected adults have shown that aerobic exercise increases VO_2_peak and resistance training increases strength and muscle size without adverse effects or immune suppression.[16,17,23,24] With regards to exercise trials conducted exclusively in older HIV-infected adults, notably there is only one study, a resistance training intervention in eleven older HIV-infected adults.[25] To our knowledge, our findings are the first to demonstrate the effect of aerobic exercise in older HIV-infected adults. We found a clinically significant increase in VO_2_peak after high-intensity aerobic training of 3.6 mL/kg/min, which represents oxygen utilization of 1 metabolic equivalent (MET; 3.5 mL/kg/min). In the general geriatric population, an increase of 1 MET over a decade is associated with 15% and 19% lower risk of all-cause and CVD mortality.[10] In comparison, exercise trials in younger HIV-infected adults have shown a 7–25% increase in VO_2_peak after 8–12 weeks of high-intensity aerobic training.[26] In this select group of older HIV-infected men, high intensity aerobic training was well-tolerated with an attrition rate comparable to the moderate intensity group (Fig 1) and overall lower (27%) than a similar randomized exercise trial in younger HIV-infected adults (32%).[18] These findings support high-intensity aerobic exercise in older HIV-infected adults.

Surprisingly, we did not find any change in fitness in the moderate aerobic exercise group (ΔVO_2_peak 0.4 mL/kg/min) despite increased exercise endurance and six-minute walk distance. The 27% increased time on treadmill in the high-intensity group was more than double the 11% increase seen in the moderate-intensity group and is consistent with the higher dose of exercise (intensity x time) received by this group compared to the moderate-intensity group, who exercised for the same duration and frequency. This dose-based increase in time on treadmill by group is similar to results in a 12-week randomized trial of moderate-intensity versus high-intensity aerobic exercise including HIV-infected men who were on average 26 years younger than our participants.[18] Numerous exercise trials in younger HIV-infected men demonstrate that 6–24 weeks of moderate-intensity aerobic training significantly increases cardiorespiratory fitness. [27–31] Results are more modest in women with HIV (Δ 1.5 mL/kg/min), but this exercise trial was home-based.[32] Our findings are noteworthy given that the intensity threshold for aerobic exercise to increase VO_2_peak in healthy adults is ≥60% of baseline VO_2_peak.[19] Accordingly, moderate-intensity exercise may not be sufficient to increase VO_2_peak in every individual, especially those young and active. However, exercise trials in older adults with low VO_2_peak and age-related diseases, including heart failure,[33] coronary artery disease,[34] chronic obstructive pulmonary disease,[35] and diabetes,[36] all report increased VO_2_peak with moderate-intensity aerobic training. In our trial, aerobic exercise at an intensity level of only 3 METS should have been sufficient to significantly increase VO_2_peak in the moderate-intensity group given the low baseline VO_2_peak (17.8 mL/kg/min).[37] Because of the small sample size, our findings need to be taken with caution and reproduced in a larger trial Nonetheless, the results raise the question of an exercise intensity threshold necessary to increase cardiorespiratory fitness in older adults with HIV, which has substantial clinical implications. The moderate-intensity exercise training in our trial meets the definition of moderate- intensity physical activity (3–6 METS) as recommended by The American Heart Association,[6] The Centers for Disease Control and Prevention (CDC),[38] and The American College of Sports Medicine.[39] Yet there is evidence that HIV-infected adults are less likely to meet even these recommendations compared to the general population.[40] Furthermore, recent exercise trials in HIV-infected adults suggest that exercise intensity[41] and loss of body fat[42] are important predictors to reduce inflammation. Our preliminary findings need to be confirmed in a larger trial with a more diverse population. Future research with measure of cardiorespiratory fitness and biomarkers of aging will address the question of whether physical activity guidelines need to be tailored for older HIV-infected adults.

In addition to the study’s small size, specific limitations warrant comment. First, the study population consisted largely of African American men which limits the generalizability of our findings. Second, since there is little information on the safety of exercise interventions in older HIV-infected adults, by design our exclusion criteria were conservative. This process resulted in a high screening to enrollment ratio but minimized the risk of serious adverse events. Therefore, the exercise safety, like the robust exercise training effects, may not be generalizable to the general population of older adults with HIV. Similarly, our attrition rate was lower than the average attrition rate in HIV exercise trials.[43] In this small pilot trial, despite randomization the groups were not balanced on weight. However, body weight does not change the response to exercise; participants in the moderate-intensity group, who were on average heavier, still improved in time on treadmill and 6-MWD, but not VO_2_peak. Even with adjustment for weight (ml/kg/min), there was no significant change in the VO_2_peak in the moderate intensity group compared to the substantial change in the high intensity group. Further, we adjusted the between group analyses for baseline differences. We acknowledge the possibility of a type II error (false negative conclusion) in the moderate-intensity group VO_2_peak finding. However, it is notable that despite the sample size statistically significant increases were found for both time on treadmill and 6-MWD, which also negates the issue of a learning effect in the high-intensity group who trained on the treadmill.

## Conclusion

This pilot randomized aerobic exercise trial provides novel findings on the safety and efficacy of moderate to high intensity aerobic exercise in older HIV-infected men. Clinically significant gains in cardiorespiratory fitness, endurance, and ambulatory function were found despite the small sample size. Results also raise the question of an absolute or relative exercise intensity threshold to increase cardiorespiratory fitness. Physical activity recommendations are a critical component of geriatric care and are yet to be formulated as part of the clinical practice of older adults with HIV. Further research is needed to determine effective exercise training strategies in older HIV-infected adults that will promote successful aging with HIV and assure an increased lifespan of quality years.

## Supporting information

S1 File Protocol. (PDF)

S2 File CONSORT 2010 checklist. (PDF)
