# Supplementary material for: A pilot randomized aerobic exercise trial in older HIV-infected men: Insights into strategies for successful aging with HIV
Source: PLoS One. 2018 Jun 12;13(6):e0198855. doi: 10.1371/journal.pone.0198855 (PMC5997336; doi:10.1371/journal.pone.0198855)
Supplement: S1 File — (PDF) [file pone.0198855.s001.pdf]

Protocol Title: A Randomized Clinical Trial of Aerobic Exercise Training in Older (50+ years) HIV-infected Adults

Sub-Title: Fitness and Longevity in Exercise (FLEX)

Principal Investigator: Kris Ann Oursler, MD, ScM

Co-Investigators: John D. Sorkin, MD, PhD
Alice S. Ryan, PhD
Les Katzel, MD, PhD
Charlene Hafer-Macko, MD, PhD
Barbara Smith, PhD, RN

Affiliation:
Geriatric Research, Education, and Clinical Center, Baltimore Veterans Affairs Medical Center, Veterans Affairs Maryland Health Care System, Baltimore, MD, USA

Department of Medicine, Division of Geriatrics and Gerontology, The University of Maryland School of Medicine, Baltimore, MD, USA

PI Contact: Krisann.Oursler@va.gov

University of Maryland IRB Protocol #: HP-00042601

ClinicalTrials.gov # NCT03399136

This research was supported by National Institute on Aging K23 [AG024896 to KKO]; Baltimore Veterans Affairs Medical Center Geriatric Research, Education and Clinical Center (GRECC) (to ASR, JDK, LIK); National Institute on Aging Claude D. Pepper Older Americans Independence Center [P30-AG-028747 to ASR, JDK, LIK, KKO]; and National Institute of Diabetes and Digestive and Kidney Diseases Mid-Atlantic Nutrition Obesity Research Center [P30-DK-072488 to ASR]; Veterans Affairs Senior Research Career Scientist Award (to ASR).

STATEMENT OF COMPLIANCE

This clinical trial will be conducted in accordance with the protocol and Good Clinical Practices (GCP) as required by the following:

- United States (US) Code of Federal Regulations (CFR) applicable to clinical studies (45 CFR 46; 21 CFR Parts 50 and 56; 21 CFR Part 312).

- ICH E6; 62 Federal Register 25691 (1997)

- NIH Clinical Terms of Award

All key personnel (all individuals responsible for the design and conduct of this study) have completed Human Subjects Protection Training.

Refer to: *http://www.hhs.gov/ohrp/humansubjects/guidance/45cfr46.htm#46.*
http://www.fda.gov/cder/guidance/959fnl.pdf
http://grants.nih.gov/grants/guide/notice-files/NOT-OD-01-061.html
http://www.cancer.gov/clinicaltrials/learning/page3

Abbreviations: Standard

AE	Adverse Event/Adverse Experience
CFR	Code of Federal Regulations
CIOMS	Council for International Organizations of Medical Sciences
CONSORT	Consolidated Standards of Reporting Trials
CFR	Code of Federal Regulations
CRF	Case Report Form
DHHS	Department of Health and Human Services
DSMB	Department Safety Monitoring Board
FDA	Food and Drug Administration
FWA	Federalwide Assurance
GCP	Good Clinical Practice
HIPAA	Health Insurance Portability and Accountability Act
HIV	Human Immunodeficiency Virus
ICF	Informed Consent Form
ICH	International Conference on Harmonization
ICMJE	International Committee of Medical Journal Editors
IEC	Independent or Institutional Ethics Committee
IRB	Institutional Review Board
ISM	Independent Safety Monitor
JAMA	Journal of the American Medical Association
N	Number (typically refers to participants)
NDA	New Drug Application
NEJM	New England Journal of Medicine
NIH	National Institutes of Health
OHRP	Office for Human Research Protections
OHSR	Office for Human Subjects Research
ORA	Office of Regulatory Affairs, DMID, NIAID, NIH, DHHS
PHI	Protected Health Information
PI	Principal Investigator
QA	Quality Assurance
QC	Quality Control
SAE	Serious Adverse Event/Serious Adverse Experience
SOP	Standard Operating Procedure
US	United States

Abbreviations: Study Specific

**ADL**	Activities of Daily Living
**AEX**	Aerobic Exercise
**AT**	Anaerobic Threshold
**BMI**	Basic Metabolic Index
**BVAMC**	Baltimore Veterans Administration Medical Center
**CD4**	Cell Count: CD4 Lymphocyte Level
**cART**	Combination Antiretroviral Therapy
**CT**	Computed Tomography
**CVD**	Cardiovascular Disease
**DXA**	Dual-Emission X-Ray Absorptiometry
**EF**	Ejection Fraction
**GRECC**	Geriatric Research and Education Clinical Center
**HAART**	Highly Active Antiretroviral Therapy
**HBV**	Hepatitis B Virus
**HCV**	Hepatitis C Virus
**HOMA-IR**	Homeostasis Model Assessment-Insulin Resistance
**hsCRP**	High Sensitivity C-Reactive Protein
**HRR**	Heart Rate Reserve
**IL-6**	Interleukin-6
**IQR**	Interquartile Range
**MET**	Metabolic Equivalent
**MVIC**	Maximal Voluntary Isometric Contraction
**NRTI**	Nucleoside Reverse Transcriptase Inhibitor
**PaCo2**	Partial Pressure of Carbon Dioxide
**PI**	Protease Inhibitor
**RCT**	Randomized Controlled Trial
**RQ**	Respiratory Quotient
**RT**	Resistance Training
**RT-PCR**	Reverse Transcription Polymerase Chain Reaction
**6-MW**	6-Minute Walk
**SPPB**	Short Physical Performance Battery
**THR**	Target Heart Rate
**TNF-a**	Tumor Necrosis Factor-Alpha
**VE**	Ventilation Efficiency
**VE/VO2**	Ventilation Efficiency Peak Oxygen Uptake
**VE/VCO2**	Ventilation Efficiency Peak Carbon Dioxide
**VO2peak**	Peak Oxygen Uptake

PROTOCOL SUMMARY

**Protocol Title:**	A Randomized Clinical Trial of Aerobic Exercise Training in Older (50+ years) HIV-infected Adults
**Phase:**	IV
**Population:**	HIV-infected men and women who are ≥50 years of age
**Number of Sites:**	Baltimore VA Medical Center, Baltimore, Maryland<br>University of Maryland SOM, Baltimore, Maryland
**Study Duration:**	5 years
**Participant Participation Duration:**	2-4 weeks of baseline testing, repeated post intervention<br>16 weeks intervention
**Description of Intervention:**	Aerobic exercise training (AEX) in center-based, supervised setting 3x week for 16-weeks
**Objectives:**	The objective of this proposal is to investigate the mechanisms by which aging affects physical functioning and aerobic capacity in HIV-infected adults, and to determine if aerobic exercise training improves functional and cardiovascular performance.
**Study Design:**	Randomized trial of 16-weeks of moderate-intensity AEX versus high-intensity AEX
**Estimated Time to Complete Enrollment:**	4 years

OBJECTIVE
The objective of this proposal is to investigate the mechanisms by which aging affects physical functioning and aerobic capacity in HIV-infected adults, and to determine if aerobic exercise training improves functional and cardiovascular performance. The central hypothesis is that high-intensity aerobic exercise training will be safe, feasible, and significantly improve aerobic capacity and functional performance in older HIV-infected adults. Results will be used to design specific exercise interventions for older adults infected with HIV. Our long-term objective is to devise effective prevention and treatment strategies that will improve and preserve function in this rapidly growing older patient population.

STUDY DESIGN
This randomized trial compares the effect of moderate-intensity aerobic exercise training (AEX) and high-intensity AEX in HIV-infected adults ≥ 50 years. Outcomes include physiologic, functional, metabolic, and body composition measures that will be assessed at baseline and 16 weeks. All participants will be HIV-infected and on stable highly active antiretroviral therapy (HAART). Most of the participants will be recruited from the ID Clinic at the Baltimore VA Medical Center (VAMC) and the University of Maryland adult HIV clinic, Evelyn Jordan Center (EJC). The sample will be ethnically diverse (Caucasian, African-American, Latino/Other), reflecting the population receiving care in local clinics. Based on the scientific aims of this study, no children will be enrolled. No exclusion criteria will be based on race, ethnicity or gender. We will use the same recruitment strategies and eligibility criteria employed to enroll older HIV-infected adults in our previous cross-sectional exercise study (Oursler et al, AIDS Research HR, 2006).

TESTING PROCEDURES
Study overview with phases are summarized in Table 1. Data for primary and secondary outcomes will be collected using standardized protocols and trained staff and are summarized in Table 2. All tests performed at baseline will be repeated in 16 weeks using similar scheduling. Tests will be scheduled at least 48 hours apart, and 48 hours after exercise training in order to control for immediate effects of exercise.

Table 1. Study Overview

**Phase 1**	**Phase 2**	**Phase 3**	**Phase 4**
Enrollment and Randomization	Testing: baseline (+/-5 weeks)	*16- weeks exercise training*	Testing: 16-weeks (+/-4 weeks)
1. H&P<br>2. ECG<br>3. GXT<br><br>Randomization:<br>1) High-AEX<br>2) Mod-AEX	Outcome measures table 2	3x weekly<br><br>Total 48 sessions	Outcome measures table 2
H&P, history and physical; ECG, electrocardiogram; GXT, graded exercise test; AEX, aerobic exercise			

PHASE 1- Enrollment
Enrollment. After providing written informed consent, the initial visit will include a history and physical exam, review of safety lab values and ECG to determine eligibility. In addition, a

graded exercise test will be completed to evaluate for sub-clinical ischemic heart disease. See section on Participant Procedures for details on informed consent, eligibility, and randomization.

Graded Exercise Test (GXT): The American College of Sports Medicine (ACSM) advocate that older individuals undergo medical evaluations and exercise testing with ECG monitoring prior to participation in high-intensity exercise. The GXT assesses the participant's ability to perform increasing intensity of exercise while monitored for myocardial ischemia, arrhythmias, orthopedic problems and other exertion-related abnormalities in gait, and pulmonary function that may interfere with participation in exercise interventions. Method: Participants without evidence of asymptomatic CAD on resting ECG will undergo a modified Bruce protocol exercise treadmill test. Individuals with ST-segment depression per Minnesota Code criteria on their exercise ECG, angina complex exercise-induced arrhythmias, or marked changes in blood pressure will be disqualified from participation and referred to their primary care physicians for further evaluation and treatment.

PHASES 2 and 4 - Baseline and Follow-Up Testing

Overview: Data for primary and secondary outcomes will be collected using standardized protocols and trained staff. Specific tests are summarized in Table 2 and described below. Additional details are provided in the procedure's SOP. All procedures will be done at baseline and after the16-week intervention with exception of baseline safety laboratory testing.

Table 2. Summary of Testing Procedures by Outcome Measures

Physiologic	Functional	Strength	Blood sampling	Body Composition
1). GXT<br>-VO2peak<br>-Anaerobic threshold	1). Six-minute walk distance<br>2). Step activity monitor<br>3). Balance and Flexibility tests<br>4). Survey	1). Isometric testing<br>- MVIC quadriceps.<br>2). Grip strength	1). Safety labs*<br>2). Metabolic<br>-lipids<br>-glucose<br>-insulin<br>3). ID<br>-HIV RNA<br>-CD4 cells<br>4). Cytokines	1). DXA<br>-Fat and lean tissue mass<br>- BMD<br>2). CT scan leg<br>-CSA, HU<br>3). CT ABD<br>-VAT and SAT<br>3). BMI<br>4). Waist/hip circumference

GXT, graded exercise test; VO2peak, oxygen utilization at peak exercise; MVIC, maximal voluntary isometric contractions; BMD, bone mineral density; CSA, cross-sectional area; HU, Hounsfield unit; VAT, visceral adipose tissue; SAT, subcutaneous adipose tissue; BMI, body mass index
*Baseline only

Physiologic Outcomes

VO2peak, Peak Oxygen Consumption, by Graded Exercise Testing (GXT): Oxygen consumption at maximum exercise effort, VO2max, is the gold standard for measure of aerobic capacity. However, our experience in conducting treadmill tests in HIV participants reflects that of other studies, where only approximately half of healthy adults are able to obtain true maximal aerobic capacity, defined as a plateau in oxygen consumption during the final stage. Therefore, as with our previous studies, VO2peak will be used as the primary measure of aerobic capacity. Method: Participants who failed to meet at least 2/3 of the criteria for peak aerobic capacity on screening GXT will perform a baseline GXT. Participants will be asked to exercise to voluntary exhaustion during a treadmill test using a modified Bruce protocol. After resting ECG and blood pressures are obtained, the test starts at 0% grade and 1.7 mph. After 2 minutes, the grade of the incline is increased to 4%, and then advanced by 2% every 2 minutes until voluntary exhaustion, or termination criteria, per the American College of Sports Medicine

recommendations (ACSM, www.acsm.org). O_2 consumption, CO_2 production, and minute ventilation will be measured breath-by-breath using a metabolic cart and values averaged for 10 second intervals. The final two 10 second values of oxygen consumption are averaged to provide the VO2peak. Analyses: In addition to directly measuring VO2peak, data on respiratory oxygen uptake, V'_{O2} and carbon dioxide outflow, V'_{CO2} will be used to estimate the respiratory gas exchange (RER= V'_{CO2}/ V'_{O2}), and to determine the oxygen exchange threshold. Data on minute ventilation, V'E, at peak and maximum values will be used to calculate breathing reserve. Oxygen pulse is an index of the stroke volume and will be calculated as VO2peak in ml/min /heart rate in beats per min to give the VO2 in ml/heart beat.

Functional Performance Outcomes

1. 6-Minute Walk Distance (6-MWD): The 6-minute walk test is a long distance walk test that reliably reflects activities of daily living and ambulatory function. In addition, it has been used safely in chronically ill patients to estimate aerobic capacity. During this test, participants are instructed to "cover as much distance as they can" over a flat 100 foot walking surface demarcated by traffic cones while timed by a stop watch. The intraclass test-retest reliability for distance covered in a 6-minute walk was 0.94, and coefficient of variation was 10.4.

2. Step Activity Monitoring (SAM): Daily physical activity will be measured using a Step Activity Monitor, a small ankle mounted step counter with a microprocessor. The number of steps walked during routine activity outside the exercise lab will be measured over a 48-hour period. This data will be used to compare baseline activity levels between intervention groups, and measure change in activity level during the study period. Participants will also be asked to keep a log of type and duration of their daily activities.

3. Balance and flexibility: The standing balance test and the flexibility task test are part of a standard functional performance testing battery. We will determine if the high prevalence of peripheral neuropathy is associated with reduced balance and flexibility, and whether exercise training improves these indices.

4. Self-reported measures- Surveys: The Fatigue Severity Scale, the Visual Analogue Scale, and the Short Form Survey Instrument [SF-36] survey will be administered to measure perceived daily fatigue, health related quality of life, and level of independent functioning. FSS score and SF-36 domains will be compared to measures of functional performance and stamina.

Muscle Strength Outcomes

1.Isometric Strength Testing: Assessment of strength will be obtained with measure of the maximal voluntary isometric contraction (MVIC) of the knee extensors (quadriceps). These tests will take place at least 24 hours after graded exercise testing on the treadmill or training session. The test session should last 0.5 - 1 hour, including set-up time. Prior to the test, participants will be educated on the purpose of the test, i.e., the strongest force that the leg muscles can create, even though the knee will stay in the same location. Participants will be seated on a specially constructed exam table with a recumbent back rest positioned at 20˚, with arms held across the chest area and with both legs hanging freely off the end of the table. A padded ankle cuff will be attached to the side being measured and linked in series with a force transducer-restraining cable, pre-set to limit knee extension at 45˚. A seat belt and thigh restraint will be used to isolate knee extensors and limit hip or trunk assistance during the tests. Up to three measures of maximum force produced during 3-5 sec. will be collected, with a 60-

sec rest period interspersed between tests. During the test, staff will verbally urge participants to "kick-out" as hard as they can for 5 sec. This series will then be repeated for the other leg. The highest magnitude force generated from the trials will be used as the measure of maximum strength of each limb.

2. Grip Strength. Bilateral grip strength will be measured three times, and kg force averaged for the dominant and non-dominant sides.

Blood Sampling: Laboratory testing:
Laboratory samples will be coded with a study ID. Venous phlebotomy will be performed for the following tests:

1. Baseline safety laboratory specimen (CBC, CMP (chem14)) will be collected on visit 1 only if they are not available in the participant's medical chart within the prior 3 months. (For the sub-study, to minimize risk of bleeding from muscle biopsy, CBC and PT values from within 4 weeks of the biopsy must be available.) This will minimize the total blood volume needed for the study.
2. Metabolic and muscle-related testing: fasting serum glucose, lipids, insulin; CPK, one EDTA tube, one citrate tube, will be collected when the participant is fasting for at least 12 hours. The serum from the EDTA and citrate tubes will be frozen and stored for testing of specific cytokines. A one-time testosterone level will be drawn at baseline.
3. HIV-related- HIV-1 RNA viral load (Roche Amplicon PCR) and CD4 cell count will be drawn with the first blood sampling on Visit 1 and Visits 7,10.

Table 3. Summary of blood sampling

Study visit	Week	Safety	Metabolic	HIV related	Total Vol. approximately:
1 or 4	0-3	X	X	X	45-60 mls
7	16-19		X	X	30-40 mls

Urine pregnancy testing:
Urine pregnancy testing will be performed on women of child bearing potential at visits 1 (screening) and prior to radiologic testing. All women will be considered of child bearing potential with the exception of women who are sterile as result of surgical procedure or have amenorrhea for six months or longer.

Body Composition Outcomes
1. Dual-energy x-ray absorptiometry (DXA) (Model Lunar Prodigy) will determine total percent body fat, fat mass, lean tissue mass and bone mineral density of the total body, hip, and spine and will characterize baseline body composition of participants.

2. Computed tomography (CT) scans using a PQ 6000 Scanner (GE Medical Systems) performed on the lower extremity every 4 cm starting at the patella to the femoral head will quantify muscle area (Hounsfield units (HU): 30 to 80), total fat area of the thigh (HU: -190 to -30), and low density lean tissue (HU: 0 to 29) of both legs. For every axial image, the total area and the quadriceps muscle group will be manually outlined to measure the cross-sectional area (CSA). The CSA of each axial slice is multiplied by the distance between slices (4 cm) and summed across slices. These values represent total and quadriceps muscle volume, and will be expressed in cm^3. CT scans at L2-L3 and L4-L5 will determine the amount of visceral and subcutaneous fat area of the abdomen. This technique has been validated previously with an average coefficient of variation of 0.78% from repeat measurement of 300 different cross-sections from different areas of the muscle.

3. Body Mass Index (BMI) is determined by height and weight. BMI = kg/m^2

4. Waist to Hip Ratio. Circumference measurements of waist and hip will be measured per procedures three times, and averaged, and ratios calculated.

5. Nutrition and Diet. Participants will complete a diet diary for 3 days/exercise phase during the training period, which will be reviewed monthly with the physiologist. A nutritionist will review with the physiologist, who will provide feedback to the participant. Since weight loss is not a study objective, total calories will not be analyzed, but monitored to assure that excess weight loss (>10%) does not occur.

PHASES 3 –Aerobic Exercise Training

Overview: All aerobic exercise training (AEX) will be performed in center-based exercise sessions 3x week under supervision for a total of 48 sessions over approximately 16-weeks. Participants will be randomized 1:1 to moderate-intensity AEX (mod-AEX) or high-intensity (high-AEX) groups.

Justification: We have designed this aerobic exercise intervention based on prior studies done by our group in older HIV-infected adults (Oursler), younger HIV-infected adults (Smith), stroke patients (Ryan and Hafer-Macko) and older athletes (Katzel) and reports in the literature. This exercise intervention also takes into consideration our experience with exercise testing in the older HIV-infected patient population: severity of aerobic capacity impairment but intact ambulatory function, inexperience with exercise training and gym equipment, and high level of motivation.

Training Procedures: Exercise physiologists will supervise all aerobic exercise training sessions. Exercise training sessions will be conducted in a gym setting at the Baltimore VAMC exercise center (Senior Exercise Research Center, SERC). Participants will be encouraged to stay weight neutral and have their weight and diet routinely assessed to meet that goal. Adverse events were evaluated by an investigator blinded to group allocation.

The intensity of the training for each individual is based on maximum heart rate (HRmax) and resting heart rate (HRrest) during the baseline treadmill test. These values are used to calculate the heart rate reserve according to the formula of Karvonen: heart rate reserve (HRR) = (HRmax – HRrest) + HR rest. The target heart rate (THR) for each training phase is calculated as a percentage of the heart rate reserve (HRR). Treadmill velocity and/or incline will be progressed as needed to meet target heart rates within the HRR intensity range at a comfortable rate for the participant. The participant's heart rate will be measured and recorded continuously during each session using a Polar HR watch. This data will be downloaded to a computer and used to monitor duration and intensity of each aerobic training session, and maintain treatment fidelity. Exercise logs will include details on exercise type, duration, time in target heart rate zone, blood pressure and perceived intensity during each training session. An exercise physiologist or study investigator will work with each participant to help adjust treadmill velocity and/or incline at a comfortable rate to meet the target HRR.

In the high-AEX group, exercise training will be performed on a motorized treadmill with occasional substitution with the elliptical machine as needed for joint pain. Initially, participants trained for 20-30 minutes at 50-60% of HRR. Duration and intensity will be increased by 10%

weekly so that within 5-7 weeks the aerobic exercise sessions lasts 30-45 minutes at 70-85% of HRR and at the end of the 16 weeks lasts 40-45 minutes at 75-90% of HRR.

In the Mod-AEX group, participants will perform a self-paced 1-mile walk (3-5 METs) on an indoor track in the same exercise center as the high-AEX group. Initial sessions will last 20-30 minutes and will be increased weekly to 45 minutes in parallel to the duration of the high-AEX group.

Strategies to reduce attrition and non-compliance: These strategies include follow-up phone calls and letters, flexible hours for exercise sessions, convenient and free parking, exercise related incentives (radios, sneakers, jerseys), and staggered financial compensation with completion bonus. These strategies were used successfully in a recently completed 16-week diet and exercise randomized trial of 55 younger HIV-infected adults (Smith, PI). Attrition rate was 9%, compared to other exercise studies in HIV-infected adults with 20-30% attrition. We expect a comparable attrition rate since we are enrolling participants who are on a stable HAART regimen, and therefore have a history of medication compliance, similar to participants in Dr. Smith's study. In addition, older HIV adults have demonstrated superior adherence to medical treatment compared to younger HIV adults.

Treatment Fidelity: As a measure of treatment fidelity, Exercise Physiology Core staff will review duration and training intensity, and HR data weekly to assure goals of sessions are on target for the intervention phase. Since HRR should improve during the course of the training program, one of the exercise training sessions may be replaced with a GXT in the exercise lab to directly measure steady state oxygen consumption and HRmax during a standardized exercise session. This will be performed to direct training and minimize risk as needed on an individual basis if participants increase their HRmax by more than 20% from their baseline HRmax.

OPTIONAL SUB-STUDY: PILOT OF MUSCLE MITOCHOBDUIAL OXIDATIVE FUNCTION

Procedure: Skeletal Muscle Biopsies will be performed in the vastus lateralis muscle of the dominant leg in interested participants to investigate the functional mechanisms underlying reduced aerobic capacity and functional performance in older HIV-infected adults. The procedure will occur at least one week before other exercise testing or beginning of training. Skeletal muscle punch biopsies will be obtained under local anesthesia (1% xylocaine) using sterile technique from the mid-vastus lateralis muscle using a 5 mm Bergström needle (Stille-Werner). The small incision will be closed with a steri-strip. Pressure will be held to the biopsy site for 5 minutes by the study staff or investigator. Participants will be observed for one hour after the procedure, during which time a heavy weight ice pack is applied to the biopsy site. Individuals will be instructed to limit vigorous activities for 24 hours after the biopsy, but will require no limitations of their usual activities. They will be contacted within 24 hours of the procedures for any discomfort. The procedure has been well tolerated by participants in several different exercise interventions.

Specimen Processing: All muscle samples are coded with ID numbers assigned by the GERI database with pre-made labels to ensure confidentiality and adequate blinding of investigators. One muscle specimen is processed in OCT mounting medium for standard histological stains, immunohistochemistry and possible in-situ hybridization studies. These stains will analyze muscle enzyme content, fiber type and capillary density. Four to five 25 mg dissected muscle specimens are frozen in isopentane pre-cooled by liquid nitrogen and stored at –70°C. One muscle sample is

utilized for SDS PAGE and / Western Blot analysis of specific contractile proteins, mitochondrial enzymes, and other proteins of interest. A second muscle sample is utilized for mtDNA quantification. The third and fourth specimen is utilized for real time RT-PCR for specific transcripts, such as mitochondrial enzymes and respiratory chain molecules. A fifth will be used for cytokine expression, such as IL-6 and TNF alpha.

POTENTIAL RISKS AND BENEFITS

Potential Risks

There are potential risks associated with some of the procedures included in this study. However, the procedures have been planned by the investigators to minimize the danger of any major complication. All medical procedures will be supervised by qualified medical personnel who will carefully monitor the participant. If new medical problems are discovered or encountered during this protocol, participants will be referred to their physicians for further evaluation and management. Risks associated with specific procedures are discussed below:

1. Exercise testing and training: The acute risk for a cardiac event during exercise testing and training is small. The AHA consensus statement on exercise standards estimates that the acute risk of sudden cardiac arrest during exercise training in adults with known cardiac disease is approximately 1 event per 60,000 hours of aerobic exercise. In our laboratory, over the past 14 years, several hundred older individuals, including many with heart disease have undergone exercise training. To date we have had 1 acute cardiac event. The primary risk is muscle soreness and injury, which will be minimized by close supervision and appropriate warm-ups.

2. Blood sampling: There is a minimal risk of bruising and infection from blood drawing.

3. Radiation exposure: For the DXA, the total amount of exposure is 20 mR (whole body = 1 mR, lumbar spine = 7 mR, hip = 7 mR, forearm = 5 mR). The CT scan will include an abdominal scan at the L3-L4 disk space and a mid-thigh scan for both legs. The total amount of exposure will not exceed the maximum amount of 40 CTDIVOL, which is approximately equivalent to 3 two-view chest x-rays or 6 single view chest x-rays.

4. Tests of functional performance and physical activity: There is a small risk that adults will fall, get chest pain, short of breath or become dizzy during these tests. Adults will be instructed to stop the test if there are any untoward symptoms such as chest pain. We have performed >100 tests of functional performance without complication. There is no risk associated with wearing the step activity monitor.

5. Strength Testing: There is a small risk of muscle strain or pulled muscles.

7. Questionnaires: There are minimal risks associated with the administration of the questionnaires. It is possible that some adults with previously undiagnosed depression or dementia will be found to have these problems. Adults with dementia will be excluded from further participation and referred for appropriate medical follow-up.

8. Confidentiality of research tests and HIV information: Confidentiality of data is of concern, particularly when information includes HIV status. The investigative team has taken extensive steps to ensure the confidentiality of the data (details, Data Management Section).

No guarantees or assurances can be given to study participants, because the results and risks of an investigation are not always foreseeable. However, every reasonable precaution will be taken to protect the well-being of study participants.

Potential Benefits

Participants will receive a thorough assessment of their functional performance and risk factors for cardiovascular disease and bone density abnormalities. They will be told of abnormal findings and with their written permission, results will be forwarded to their health care providers for appropriate medical follow-up. In prior studies, these types of assessment have detected previously undiagnosed problems (i.e. hyperlipidemia, diabetes, osteoporosis, etc.). Participants are given surveys to self-evaluate behaviors which may also allow them to identify risky behaviors they may consider changing in the future. Participants may benefit from the exercise training, which should improve their level of functioning, exercise capacity, and potentially decrease their risk for future cardiac events. Some Participants may embrace the new behavior of exercise after the study and incorporate the exercise or increased physical activity into their routine. Any of these changes in the future will give them an opportunity to improve their quality of their life. We believe that the benefits associated with this study will exceed the risks, thereby resulting in a low risk: benefit ratio for any participant.

PARTICIPANT PROCEDURES

Privacy: Participant privacy will be maintained and protected. All research interactions, for example consent procedure, questionnaires, and examinations, will be performed in private areas (clinic or research rooms with limited traffic and ability to have conversation which is not overheard) or private rooms (closed door without others present). Research procedures will be performed in research areas with proper screens / curtains to ensure privacy. The exercise training sessions will be conducted in an open gym setting at the VA Senior Exercise Research Center where many exercise intervention studies are ongoing. Participants from all the studies are treated per protocol with same attention from staff. Since this study is limited to HIV-infected participants, great care is taken to not identify which study the participant is enrolled in.

Recruitment: Recruitment will take place with referral from the patient's medical provider, and introduction to research staff, and will take place in private area or room. After initial introduction, a member of the research team will direct the interested patient to a private room in the clinic area or the research unit to provide more information about the study.

Screening: Screening of clinical medical records for interested patients will occur by a clinical investigator with pre-HIPAA waiver to determine if the individual meets basic criteria of HIV infection, receiving HAART, and 50+ years of age.

Eligibility: Signed informed consent and signed HIPAA PHI authorization will be obtained at the first study visit after recruitment and screening. After informed consent is obtained, a history and physical examination by a clinician investigator will be performed. Hospital medical records will be reviewed and will include: lipids, glucose, liver functions, electrolytes, complete blood count, a history of medical comorbidities, AIDS defining illness, and HAART use. Specific eligibility criteria include objective cut-off limits designed to maximize safety and minimize attrition but not prohibitively limit enrollment (maintain generalizability). These eligibility criteria are based on our research in the target population, the literature, and the ACSM Resources for Clinical Exercise Physiology. This study will be inclusive of all HIV-infected patients 50+ years of age who meet the eligibility criteria, regardless of religion, sex, or ethnic background. An overview of inclusion and exclusion criteria is provided below. Specific operational criteria can be found in the eligibility checklist (appendix A).

Inclusion Criteria

1) HIV-infected adult 50 years of age and older under outpatient care for their HIV

2) Antiretroviral Therapy: Patients will be required to be on the same antiretroviral therapy for at least six months. This criterion minimizes confounding from acute medication side effects. In addition, patients who are not receiving antiretroviral therapy are very heterogeneous with a wide range of disease progression (non-progressors to hospice). Drugs that significantly affect mitochondria are no longer commonly used and will not be a confounding factor. Over 85% of the ID Clinic patients are receiving antiretroviral therapy. This proportion may increase further as criteria to start therapy earlier are gaining support.

Exclusion Criteria
1) AIDS: Patients with a recent (< 6 month) history of an AIDS-defining condition (Centers for Disease Control criteria) will be excluded to minimize the effects of disease stage on study outcomes. However, it should be noted that many of the older HIV-infected patients in the HIV Clinic have been HIV-infected for 10 years or longer. Approximately a third of our participants in prior exercise studies have had a distant history of AIDS illness prior to effective therapy. Therefore, patients with prior AIDS illness will not be excluded as they represent the target population.

2) Comorbidities: Patients with comorbid conditions that could have a potential impact on their ability to perform exercise testing and training will be excluded according to the American College of Sports Medicine. The screening evaluation focuses on comorbidities that could impact the participant's ability to perform physical work including, but not limited to: hemiparetic stroke, dementia, neuropathy, falls, peripheral vascular disease, coronary artery disease, severe pulmonary disease, cancer treatment, steroid treatment, end stage liver disease, and chronic renal failure. Anemia is common in HIV-infected patients. We will use a cutoff of hemoglobin <10 g/dl to exclude patients, based on our findings of the association of anemia with low VO2peak in the target population (Oursler et al, AIDS Research HR, 2006). Current smoking will not be an exclusion criterion. Our preliminary data show that 60-70% of the otherwise eligible HIV-infected patients are smokers. Therefore, it is not feasible to exclude smokers.

3) Activity Level: Only sedentary adults will be eligible. Individuals that participate in regular structured aerobic exercise or resistance training in the prior 6-months will be excluded. Our preliminary data show that the majority of HIV Clinic patients are sedentary.

Informed Consent and HIPAA: Written informed consent will be obtained from each participant in a private room. The procedures will be discussed in detail at the first visit with the participant by the research staff or PI. Informed consent will be obtained by the PI, co-investigators, or research staff. The participant will be provided a copy of the informed consent form and the HIPAA. The signed consent forms and HIPAA are kept in a locked filing cabinet in the study's research office at the Baltimore VA Medical Center. If the participant is a Veteran, a progress note will be written documenting the informed consent process will be placed in the VA computerized patient record system (CPRS) system per VA requirements. The participant will receive a series of detailed appointment letters that confirm the procedures for the next scheduled research visit. Participants will also receive reminder calls prior to most scheduled visits during which they are reminded of the upcoming procedures and given the opportunity to ask questions. The research staff is also present during each visit to answer questions, and they will be free to withdraw at any time. In accordance with good clinical practice guidelines, participants will be informed on clinically significant findings. Clinically significant findings will be communicated to their predesignated primary care provider for clinical follow-up.

HIPAA statement will be reviewed and signed at the time of the informed consent, and will be stored with the informed consent form. A partial waiver of HIPAA is requested for screening purpose only. No study procedures, including history and physical exam by the research team, will occur prior to written consent and completion of the HIPAA form.

Randomization Procedures: Patients that meet eligibility criteria and successfully complete baseline testing will be randomized 1:1 to moderate-intensity or high-intensity groups using the closed envelope method. That is, prior to enrollment of any participants, participant numbers will be assigned to either group based on a computer random assignment procedure and placed in small sealed pre-coded envelopes by Dr. Sorkin Baltimore VA GRECC Biostatistics Head, who does not have participant contact. Randomization will occur in blocks of 10 individuals. This method is designed to ensure that one treatment group does not end up with an unbalanced number of participants in smaller trials. This means that 10 individuals are randomized 1:1 across groups and placed into an envelope at one time. Dr. Sorkin will assign a participant to a group by selecting the next sealed envelope and giving it to the research coordinator, who will request it at the end of phase 1. Since approximately 10% of HIV-infected adults may have undiagnosed CAD, phase 1 includes GXT prior to randomization.

Procedures for Blinding: Since it is not feasible for intervention to be blinded or masked, only study personnel who do not participate in the exercise training will perform the study assessments related to exercise and physical function performance. Research staff who have no participant contact, or access to the baseline data, will oversee the randomization procedure.

Participant Withdrawal/Discontinuation: Participants are free to withdraw from participating in the study at any time upon their request. Participants who elect to withdraw from the study will not perform additional research portions of the study (i.e., research blood draws). Data that has been already collected prior to withdrawal will be used in analysis, unless the participants request otherwise in writing. A study participant will be discontinued from active participation in the study (and followed for safety) if:

- Any adverse event (AE), laboratory abnormality, inter-current illness, or other medical condition or situation occurs such that continued participation in the study would not be in the best interest of the participant.
- Develop any exclusion criteria is cause for discontinuation.

Compensation: Participants will be reimbursed weekly either by an electronic funds transfer (EFT) to their bank account or by check, according to their preference. Baseline (phase 1) and follow-up (phase 3) testing are reimbursed at a rate of $20/test. During the 16 weeks of the Exercise Program (Phase 2), participants are reimbursed $5 per exercise session that is attended. If the study staff finds that that an additional visit is required to repeat a test or blood draw, reimbursement will be at a rate of $20 per visit.

DATA MANAGEMENT

Quality Assurance Procedures

The investigational site is responsible for conducting routine quality assurance (QA) and quality control (QC) activities to internally monitor study progress and protocol compliance. The Principal Investigator will provide direct access to all study-related materials, source data/documents, and reports for the purpose of monitoring and auditing by the sponsor, and inspection by local and regulatory authorities. The Principal Investigator will ensure all study personnel are appropriately trained and applicable documentations are maintained on site.

VAMHCS Office of Research Compliance (ORC) monitors will assess that the study is conducted, and data are generated, documented (recorded), and reported in compliance with the protocol, Good Clinical Practice, and the applicable regulatory requirements. Clinical monitoring reports will be submitted to VA R&D and the IRB. The Principal Investigator will implement quality control procedures beginning with the data entry system and generate data quality control checks that will be run on the database. Any missing data or data anomalies will be communicated to the site(s) for prompt clarification and resolution.

Research Records
The PI is responsible to ensure the accuracy, completeness, legibility, and timeliness of the data reported. Forms for use as data collection forms will be derived from the case report forms (CRFs) to record and maintain data for each participant enrolled in the study. All data collection forms should be completed in a neat, legible manner to ensure accurate interpretation of data. Black or blue ink is required to ensure clarity of reproduced copies. When making a change or correction, cross out the original entry with a single line and initial and date the change. Do not erase, overwrite, or use correction fluid or tape on the original. Data reported in the CRF derived from data collection forms or source documents should be consistent or any discrepancies should be explained.

Confidentiality
The study protocol, documentation, data and all other information generated will be held in strict confidence. In order that participant confidentiality can be maintained, all records will be kept in locked rooms and cabinets, and on secure password protected computer servers. The following confidential information will be collected and stored separately from study data in a safe, locked location: date of birth, name, social security number, full address, phone number and emergency contact name and phone number. Results of testing (data) and case report forms will be coded with a study ID only to prevent association with PHI. Blood samples will be collected and stored with code numbers as the only identifiers. Laboratory assays containing data from the study will be labeled only with code numbers. A master list with key code between PHI and study ID will be maintained by a single research coordinator with access only to the PI, VAMHCS Office of Research Compliance (ORC), VA R&D and the IRB. The results of the research study may be published, but participants' names or identities will not be revealed. Records will remain confidential. No information concerning the study or the data will be released to any unauthorized third party, without prior written approval of the sponsor and the IRB. When study personnel are no longer part of the research team, we will amend the data access request to terminate that person's access to all study data and notify the VA Information Security Officer of such action. No sensitive patient data will be shared with anyone who does not have a VA appointment. All study team personnel with access to sensitive patient data will stay current on required VA information security and privacy policy trainings.

Storage of data will be HIPAA compliant. Written records will be stored in locked research offices at the Baltimore VA Medical Center with limited access to PHI as described above. Research data collected by the University of Maryland Division of Gerontology is stored in GERI. GERI is a relational database that conforms to open database connectivity (ODBC) standards, and is behind the VA firewall. GERI is maintained by the biostatistics cores of the Baltimore VA Medical Center GRECC and University of Maryland Pepper Center. Access to the GERI database is limited to authorized individuals. All computers that can access the database are located in locked offices, and are protected from unauthorized access by a password. Once the computer is unlocked, two further passwords are required to access the database. Network software ensures that each user change his passwords at least once every six months. To

protect against external attack, the database can only be accessed by a computer attached to our local area network; web access is not allowed. The list mapping the study ID to enrolled/consented patients and their hospital record number will be in a specific table in GERI, separate from the rest of the study data. Only Drs. Oursler and Sorkin and the primary study coordinator will have a password and access to this table in GERI. Some of the results of the research tests performed at the Baltimore VA Medical Center will be stored on the VA's CPRS (EMR). This includes participant enrollment notes, documentation of the informed consent process, and electronic scanned copies of the consent forms. CT scans and DXA scans (raw data) will be stored digitally on the Baltimore VA Medical Center PACS system that will permit subsequent image processing. Although CPRS and the PACS are double password protected computer information systems, the results stored in the CPRS and PACS systems could be accessed by VA clinical personnel who are not affiliated with this research project.

Data Retention

Records and documents pertaining to the conduct of this study, including CRFs, data collection forms, consent forms, test results, and lab results, must be retained by the investigator for at least 3 years following closing of the study. Destruction of study records will be performed per VA policy.

Appendix A: FLEX Main Study
Inclusion/Exclusion Criteria Checklist

Yes	No	Inclusion Criteria
		1. HIV-infected
		2. Age: 50 years and older
		3. Stable HAART regimen (prescribed for 6 months or longer).

Yes	No	Exclusion Criteria
		1. a recent history of AIDS defining illnesses (within 6 months)
		3. myocardial infarction (within 3 months)
		4. exertional or unstable angina (chest pain that limits activity)
		5. severe congestive heart failure (EF < 20% in last year or NYHA Classification III or IV)
		6. uncontrolled hypertension (SBP over 180 &/or DBP over 110 mm Hg, any single measure at screening)
		7. screening EKG with ischemia, complex arrhythmia, or high grade conduction defect (Minnesota Code)
		8. poorly controlled diabetes mellitus (FBS>200 mg/dl, RBS > 299 mg/dl, or HbA1C > 10; < 1 month)
		9. treatment for cancer (within 3 months)
		10. peripheral Vascular Disease with claudication
		11. severe arthritis limiting ambulation
		12. neurologic disease limiting ambulation (stroke hemiparesis)
		13. end stage liver disease (decompensated liver disease)
		14. chronic renal failure (dialysis)
		15. anemia (HGB <10)
		16. severe pulmonary disease (home O2, admission for dyspnea or pneumonia within 1 month)
		17. treatment with anabolic steroids or glucocorticoid steroids or growth hormone (within 3 months)
		18. dementia (based on Evaluation to Consent)
		19. pregnant (negative urine testing at screening for women of child bearing potential; check no for others)
		20. signs or symptoms of any medical comorbidity that would preclude exercise testing or training
		22. therapy with b-blocker or non-dihydropyridine calcium channel blocker (within 1 month).
		25. history of known skeletal muscle disease.
		26. exercising on a routine basis (strength training or aerobic exercise more than 1 times per week).

_______________________________________ ____/____/____

Initials of Subject Date Screened

Reviewed and subject acceptable for study: ___YES ___NO

_______________________________________ ____/____/____

Signature of Principal Investigator Date
